# Anxious or empowered? A cross-sectional study exploring how wearable activity trackers make their owners feel

**DOI:** 10.1186/s40359-019-0315-y

**Published:** 2019-07-03

**Authors:** Jillian Ryan, Sarah Edney, Carol Maher

**Affiliations:** 1 Commonwealth Scientific and Industrial Research Organisation, Gate 12 Kintore Avenue, Adelaide, South Australia Australia; 2 Alliance for Research in Exercise, Nutrition, and Activity, University of South Australia, GPO Box 2471, Adelaide, South Australia Australia

**Keywords:** Physical activity, Wearable activity tracker, Affect, Personality, Behaviour change

## Abstract

**Background:**

The market for wearable activity trackers has grown prolifically in recent years, with increasing numbers of consumers using them to track, measure, and ideally improve their health and wellbeing. Empirical evidence tends to support wearables as valid, reliable, and effective health behaviour change tools, however little research has been conducted to understand experiential aspects of the devices, particularly thier effects on users’ psychological wellbeing and affect. This study addresses this literature gap by exploring wearable users’ affective responses to their devices and how these relate to personality traits and individual differences.

**Methods:**

Data were collected from adult wearable users (*N* = 237) via an online survey that assessed participant demographic characteristics, personality trait profiles, and experiences of negative (guilt, self-consciousness, & anxiety) and positive affect (empowerment, motivation, & accountability) related to their wearable both during wear, and when unable to wear (e.g. if the battery ran flat). Outcomes were analysed descriptively and general linear models used to examine associations between affect scores with personality traits and individual differences.

**Results:**

Both current and previous wearable users experience more positive than negative affect related to their device whilst they were wearing it (*p* = <.001). When prevented from wearing their device, however, this pattern was reversed with most participants reporting stronger negative than positive affect (*p* = <.001). These patterns were generally consistent across demographic sub-groups and personality trait profiles, although conscientiousness and openness to experience were independently and positively associated with affect during wear (*p* = .001).

**Conclusions:**

Results suggest that using a wearable is a positive experience for users with little risk of negative psychological consequences. Whilst experiences of negative affect were uncommon, individuals low in conscientiousness or openness to experience are at greater risk of negative affect and all users may experience negative affect such as anxiety or frustration when prevented from wearing their device. Findings contribute to mounting evidence of wearables’ safety and appeal as health behaviour tools and highlight the importance of examining psychological and experiential aspects of activity tracking.

## Background

Despite significant public health promotion efforts, physical inactivity remains to be a major risk factor for lifestyle-related chronic diseases including heart disease, type 2 diabetes and cancers, as well as reduced quality of life and poor mental health, particularly in developed countries [1]. Novel and effective behaviour change tools are needed to assist individuals to adopt healthier lifestyles. One such tool is the wearable activity tracker; a small, typically wrist-worn device that measures and tracks health and activity information including daily step and stair counts, minutes of activity and sedentary behaviour, sleep quality and quantity, and heart rate [2], with information displayed to the user via the device itself and an accompanying smartphone application [app] or website. Wearables have spread prolifically throughout the consumer market in recent years, with their efficacy as health behaviour change tools resting on the premise that users will use the objective health and activity information provided by their wearable to alter their behaviour, similar to biofeedback [3].

There is an emerging body of evidence to suggest that wearables can lead to positive, albeit modest, effects on users’ physical activity levels (see reviews, [4, 5]). The reliability, validity, and content of the devices themselves has also been studied with results indicating moderate to strong measurement reliability and validity (particularly for step counts, [6]) and the presence of evidence-based behaviour change techniques such as social comparison/support and positive reinforcement (e.g. virtual gifts or badges) to reward desired behaviours [7], particularly for physical activity rather than sleep or sedentary behaviours [8].

A notable gap in the literature concerns the user experience and users’ psychological responses to their wearable activity trackers. The mainstream media commonly purports that wearables are harmful to user’s psychological health and wellbeing (e.g. [9–12]). Well-respected news outlets including BBC News and The Conversation, for example, have both described a ‘dark side’ to wearables, arguing that they lead to obsessive tendencies, rumination, and anxiety in users who worry about checking their data or meeting their daily goals [9, 10]. An article published in ‘The Conversation’ reported results of an survey that indicated that users experienced feelings of guilt and pressure to achieve their daily goals, with some experiencing such intense negative emotions that they viewed their wearable as an ‘enemy’ [10]. Assertions that wearables are detrimental to users’ psychosocial health are supported by little empirical evidence, however, since there has been minimal research on this topic.

Just one peer-reviewed study has examined experiential aspects of wearable activity trackers amongst typical [i.e., non-clinical, adult] users [13], to the best of our knowledge. In that study users [*N* = 133] were asked to describe a memorable experience that they had had with their wearable and to rate the extent to which that experience fulfilled each of ten psychosocial needs, for example; “autonomy (i.e., feeling that one is the cause of his or her own actions rather than feeling that external forces or pressure are the cause of his or her action) and competence (i.e., feeling that one is very capable and effective in his or her actions rather than feeling incompetent or ineffective)” [13]. Analyses revealed that the wearables fulfilled needs of physical thriving, autonomy, and competence and suggested that participants derived multiple psychological and social benefits from their devices, in addition to physical benefits.

Contrary to findings from Karapanos et al. [13], broader experimental evidence suggests that self-monitoring may be associated with negative psychological consequences [14–16], which may also apply to wearable activity trackers. This was demonstrated by one study that randomly allocated college students (*N* = 91) to either wear a standard pedometer or a pedometer that had an obscured display [16]. Participants were instructed to report back at the end of a day of free-living conditions to report their step counts and the extent to which they enjoyed walking. Results showed that participants who had worn a standard pedometer registered higher step counts but lower enjoyment of walking compared to those who had worn the display-obscured pedometer. This response pattern was replicated with colouring and reading tasks, indicating that self-monitoring can elicit faster performance and reduced enjoyment regardless of the nature of the activity, described by the author as “the hidden cost of personal quantification” [16].

The aim of the current study is to address the previously described research deficit by examining wearable users’ affective responses to their devices and the relationships between these and user characteristics including personality traits. The type and intensity of affect (state-based emotion) experienced during and shortly after an activity may determine future behaviour via positive and negative reinforcement and serve as an indicator of the activity’s effects on the individuals’ psychosocial wellbeing [17]. Therefore, it is important to consider how wearables alter users’ affective states during wear and when they are prevented from wearing their devices, since this may contribute to how they feel about physical activity, the likelihood that they will continue to perform physical activity, and their psychosocial well-being in general. Based on previous research, it could be expected that affective responses to wearables may be modulated by users’ characteristics such as their socio-demographic characteristics and personality trait profiles [18]. Conscientiousness and neuroticism, for example, are known to play an important part in determining health behaviours due to their role in self-regulation, self-discipline, and propensity to experience negative emotion [19]. Due to the limited amount of existing research in this area and the exploratory nature of the study it was not possible to formulate hypotheses, however, the following research questions are proposed:

1. What are users’ affective responses to their wearable both during wear and when unable to wear?
2. How are affective responses in relation to wearables associated with participant socio-demographic characteristics and personality trait profiles both during wear and when unable to wear?
3. How do current and previous users differ in their experience of affective responses to their wearable?

## Methods

Data used in this study were collected as part of a larger cross-sectional survey that explored the user experience with wearables including device usability, technical issues, and perceived behaviour change [20]. The study had ethical approval from the University of South Australia Human Research Ethics Committee and all participants provided informed consent prior to participating.

The primary aim was to assess affective responses to wearable activity trackers amongst current users’ of activity trackers, as implications of the results are likely to affect this population the most closely. A secondary aim was to investigate differences in affective responses to wearable trackers between current users and previous users who had ceased using their activity trackers for one reason or another.

### Participants

Eligibility criteria required that participants be Australian residents, at least 18 years old, and current or previous user of a wearable activity tracker that captured at minimum daily step counts and was ‘smart’, that is, had capacity to share data to an external website or smartphone app. Participants were recruited to the study via a low-cost, Facebook-delivered recruitment campaign. Specifically, we identified a variety of Facebook community groups that represented audiences across Australia and shared a post that advertised the study to those groups. As incentive to participate and on-share recruitment posts on Facebook, participants had the opportunity to enter into a draw to win a $50 online voucher to a sporting goods store, with one draw offered for sharing a link to the survey on Facebook and another draw offered upon completion of the survey. The online survey was completed by 237 participants including 200 current users and 37 previous users. Demographic data is displayed in Table 1 and described in the results section. As reported elsewhere [20], users had worn their activity trackers for a mean duration of 7 months (range 0 - > 36 months) and the most commonly used brand of activity tracker was Fitbit (68% of users) followed by Garmin (17% of users) and Apple watch (3.4% of users).

**Table 1:** Participant demographic and personality characteristics

	Current wearers (*N* = 200)	Previous wearers (*N* = 37)		
	n	% of N	n	% of N
Sex (female)	141	71%	27	73%
Education level				
High school or less	40	20%	12	32%
Trade or vocational training (e.g. certificate, apprenticeship)	38	19%	4	11%
University degree	122	61%	21	57%
	M	SD	M	SD
Age (M ± SD)	33.67	12.34	29.11	± 12.11
Personality trait scores				
Extraversion	M: 4.55	SD: 1.48	−^a^	−^a^
Agreeableness	M: 4.93	SD: 1.08	−^a^	−^a^
Conscientiousness	M: 5.20	SD: 1.26	−^a^	− ^a^
Neuroticism	M: 3.08	SD: 1.29	−^a^	−^a^
Openness to experience	M: 4.99	SD: 1.08	−^a^	− ^a^
Occasions per day data checked	M: 6.17	SD: 4.61	−^a^	−^a^
Affect during wear^b^	M: 3.98	SD: 0.53	3.64	0.71
Affect when unable to wear^b^	M: 2.69	SD: 0.56	2.77	0.67

^a^Data regarding personality traits and tracker usage behaviour was collected from current participants only. Personality trait scores range from minimum 1 - maximum 7 and higher scores indicate stronger endorsement of each trait

^b^Affect scores range from minimum 1 – maximum 5 and higher scores indicate more positive affect

### Scope of the survey instrument

The survey contained 26 items, including a combination of existing and purpose-designed measures, and was compiled using a rigorous process including consultation with three experts in the field who confirmed face validity as well as pilot testing within the target demographic.

#### Socio-demographic characteristics and frequency of checking data (4 items)

Four items assessed participants’ socio-demographic characteristics including sex, age, and education level [high school or less, technical or further education institutions [e.g. TAFE], university degree] and how many times per day they checked their wearable data.

#### Personality trait profiles (10 items)

The Ten Item Personality Inventory [TIPT] [21] was used to assess current users' personality trait profiles. We did not assess the personality trait profiles of previous users to minimise burden for this group, who were not a primary focus of this study. The TIPI assesses the extent to which respondents endorse each of five personality traits: extraversion, agreeableness, conscientiousness, neuroticism, and openness to experience. Each item asks participants to indicate the extent to which a pair of descriptors (e.g. “anxious, easily upset” or “critical, quarrelsome” [neuroticism]) applies to them, with responses recorded on a 7-point Likert scale. The four negatively worded items are reverse-coded prior to data analysis such that higher summed scores reflect stronger trait endorsement. The TIPI is a preferred tool for brief personality assessments that has been shown to have adequate psychometric properties including moderate test-retest reliability coefficients (0.72 over 6 weeks) and moderate convergent validity coefficients with the Revised NEO Personality Inventory of 0.63 [22]. Internal consistency of our data was assessed using Spearman-Brown coefficients due to each personality trait subscale containing just two-items. Spearman-Brown coefficients for individual trait subscales varied, specifically, Extraversion r_s_ = 0.53, Agreeableness r_s_ = 0.23, Conscientiousness r_s_ = 0.50, Neuroticism r_s_ = 0.50, and Openness to Experience r_s_ = 0.33.

#### Affective responses to wearables [10 items]

A ten-item scale was developed to assess users’ affective responses to their wearables. Similar to existing instruments (e.g. Positive and Negative Affect Scale, [23]), these questions asked participants to indicate the extent to which they agreed that they had experienced a particular affective state or emotion in a specified time frame, with responses measured on a 5-point Likert scale. Six items were phrased to capture affect experienced during wear and four items were phrased to capture affect experienced when unable to wear. To minimise participant burden we purposefully assessed affect during wear in greater detail (six items compared to four when unable to wear) since this is likely to reflect the majority of time spent interacting with wearable activity trackers. Prior to calculating overall scores for each of the two subscales, items related to negative affective states such as guilty, self-conscious, anxious, and frustrated were reverse coded such that a higher score represented a more positive affective state. Scores on each sub-scale were summed and averaged to produce scores that ranged from 1 to 5 with higher scores reflecting a more positive affective state. The two sub-scales were:

- **Affect during wear (6 items total):** six items that asked participants to indicate the extent to which ‘when I am using my wearable it makes me feel [*empowered, motivated, accountable, guilty, self-conscious, anxious*]’.
- **Affect when unable to wear (4 items total):** four items that asked participants to indicate the extent to which ‘when I’m not using/forget/can’t use my wearable it makes me feel [*liberated*, *guilty, frustrated, anxious*]’.

Cronbach’s alpha was used to assess internal consistency of affect scales including affect during wear (*α* = .61) and affect when unable to wear (*α* = .67).

### Procedure and data analysis

Recruitment and data collection occured over a 6-week period, after which all data were downloaded into statistical software package SPSS [IBM, v21]. Data were screened for outliers and normality prior to analysis. Distribution data were examined to confirm neither that skewness nor kurtosis exceeded 1.96 times its standard error indicating normal distributions. Examination of studentized deleted residuals confirmed an absence of outliers. Participant socio-demographic characteristics and frequency of checking data were analysed descriptively. One-way ANOVAs conducted via the General Linear Model procedure assessed associations between current users’ affect scores during wear and participant characteristics. Two models were tested in total with two dependent variables: (1) affect during wear and (2) affect when unable to wear. The eight independent variables were sex, education, age, and the five personality traits. An alpha of 0.05 denoted statistical significance and exact *p*-values are reported. Given that this was a secondary analysis of an existing dataset and hypothesis-generating in nature, formal power calculations of the sample were not undertaken.

## Results

Participants were 200 current users and 37 previous users of a wearable activity tracker (see Table 1). The majority of participants (72%) were female and had completed a university degree (60%). Participants’ average age was 33.1 (*SD* 12.4) and participants checked the data on their device 6.2 (*SD* 4.6) times on average per day.

### Self-reported affect relating to activity tracker

**Fig. 1:**
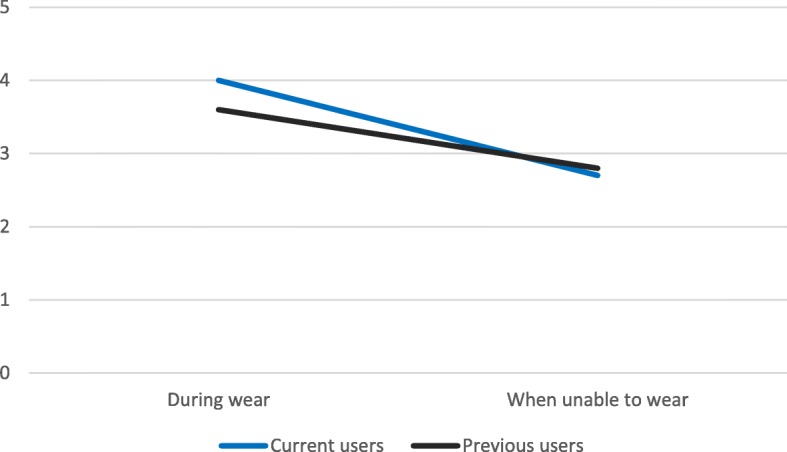
Users’ mean affect scores during wear and when unable to wear

Two-way ANOVA results indicated that affect was significantly associated with wearing conditions (previous or current user; during wear and when unable to wear), F (3, 460) = 182.152, *p* = < 0.001. There were statistically significant associations between affect scores with current or previous wear status (*p* = 0.036), wearing or unable to wear status (*p* = 0.004) and an interaction between current or previous wear status and wearing or unable to wear status (*p* = 0.004). This interaction effect is illustrated in Fig. 1. Previous and current users’ self-reported affect scores followed a similar trajectory, whereby affect was higher (more positive) during wear but declined significantly when not wearing (see Fig. 1).

### Associations between affect, and demographic and personality trait characteristics

Associations between affect scores, demographic characteristics and personality trait profiles for current users only were examined using the General Linear Model procedure in SPSS (Table 2). Affect scores when able to wear the activity tracker were positively and significantly associated with both openness to experience and conscientiousness personality traits, but unrelated to sex, age, or education. Affect scores when unable to wear the activity tracker were not associated with any sociodemographic or personality characteristics.

**Table 2:** General Linear Model displaying associations between affect scores and participant characteristics

	During wear			When unable to wear		
	*B*	*SE*_*B*_	*p*	*B*	*SE*_*B*_	*p*
Intercept	2.79	0.34	<.001	3.04	0.39	<.001
Sex						
Male	−0.03	0.08	.753	−0.27	0.09	.771
Female	-^a^	-^a^	-^a^	-^a^	-^a^	-^a^
Education						
High school or less	0.11	0.10	.274	0.06	0.12	.582
Vocational training (e.g. trade school)	0.96	0.10	.333	−0.83	0.11	.461
University degree	-^a^	-^a^	-^a^	-^a^	-^a^	-^a^
Age	0.01	0.01	.883	0.01	0.01	.524
Openness to Experience	0.09	0.04	.018*	−0.03	0.04	.574
Conscientiousness	0.07	0.03	.026*	−0.01	0.4	.871
Extraversion	0.03	0.03	.300	0.01	0.03	.831
Agreeableness	0.05	0.04	.198	−0.04	0.05	.412
Neuroticism	−0.02	0.03	.620	−0.41	0.04	.267

Note: ^*^denotes significant finding at *p* = < 0.05; ^a^ denotes redundant category

### Differences in affect scores between current and previous users

During wear, current users’ affect scores (*M* = 4.01, *SD* = 0.53) were higher (more positive) than previous users’ scores (*M* = 3.65, *SD* = 0.71) and these differences were statistically significant *t* (229) = 3.348, *p* = <.001. When unable to wear, affect scores were slightly lower for current users (*M* = 2.70, *SD* = 0.56) compared to previous users (*M* = 2.78, *SD* = 0.70) but these differences were not statistically significant *t* (230) = − 0.762, *p* = .447.

## Discussion

This study set out to investigate users’ experiences of positive and negative psychological affect in response to their wearable activity trackers, and whether these experiences and usage patterns were associated with personality and socio-demographic characteristics. Consistent with previous research in this area [13], findings indicate that overall, using a wearable is a positive experience for users with the devices being a source of multiple psychological benefits for users and few negative psychological implications. Interestingly, these findings contrast with one perspective that is commonly purported in the grey literature and mass media coverage that wearable usage leads to negative experiences and feelings of anxiety and guilt [9, 10]. In the current study, reports of negative affect associated with using a wearable were relatively uncommon, though this was more likely amongst participants who were low in conscientiousness and for all participants when they were unable to use their wearable.

While results generally reflect positively on wearables, it is important to acknowledge individual differences in how humans respond to different aspects of their environments [24], including feedback provided by wearables as revealed by findings from the current study. Related research shows that activity tracking can have negative effects on users’ psychological wellbeing, for example, reduced enjoyment of walking-based physical activity under randomised controlled trial conditions [16] and greater experiences of eating disorder symptomology amongst college students [15]. Current users showed significantly higher positive affect relating their wearable activity tracker than previous users, suggesting that these affective responses may have contributed to former users’ decisions to cease using their activity tracker. Although the current study found little evidence of negative psychological effects, analyses did show that low-conscientious participants were at greater risk of experiencing negative affect. This is consistent with evidence that individual differences affect psychological responses to aspects of the environment [18]. A potential explanation for this finding may be related to the fact that high-conscientious individuals are more likely to display an internal attribution style and devote significant attention to goal attainment [25, 26]. Wearables typically promote performance-based goals (e.g. 10,000 steps or ten flights of stairs per day) that are scored based on success or failure and it is possible that this is a source of anxiety for low-conscientious users. These users would potentially benefit more from progress- or improvement- based goals and feedback. Further research is needed to explore the effect of goal type on affective responses amongst wearables users. No associations were found between neuroticism and affect, which may have been unexpected given the effect of neuroticism on self-regulation and experience of negative emotion [19]. This may be attributable to the low mean and homogeneity of neuroticism scores within this sample.

Key contributions of this study are its novelty in this field, since it addresses a significant gap in the literature and contributes to the expanding body of evidence suggesting that wearables are useful and positive health behaviour change tools. Study strengths include the use of an established tool to measure personality characteristics and that the reliability of purpose-designed questionnaire items was scrutinised. A further strength of the study was that it examined the positive and negative experiences of both current and former users, rather than focussing solely on one user group. In terms of limitations, it is important to recognise self-selection biases as a limitation to internal validity and the generalisability of findings to other population groups, for example individuals who are prescribed a wearable in clinical intervention scenarios. Furthermore, the sample is skewed towards females (73%) and current users (84%), who may be more interested in answering questions about their wearable and may have a greater likelihood of experiencing positive affect. In addition, some of the personality scales, such as Agreeableness and Openness to Experience showed low internal consistency, which calls into question the reliability of these data. It is also worth noting that the original study was designed to be descriptive in nature, and that this study was hypothesis generating, thus formal power analysis/a priori sample size targets were not set. However, we acknowledge that the sample size is modest, and therefore it is likely that the study may be underpowered to detect some relationships that are actually present in the data set. The affect scales that we used in this study were purpose-designed and adapted from existing scales. There were some limitations associated with this, including modest internal consistency. Unfortunately, few validated scales exist that specifically assess consumer perspectives of new health technological devices, which is an important avenue for future work.

Further research is warranted to examine the psychological impacts of wearable activity tracker usage amongst younger users, those with higher levels of education and those with higher levels of conscientiousness, as these groups were highlighted as being at increased risk of negative affect. One research question that warrants further exploration is whether certain individuals’ experience with wearables could be improved through use of progress-based rather than performance-based goals. In addition, researchers have also pointed to the design features of wearables as a key determinant of their efficacy as health behaviour change tools, which also warrants further investigation [27]. Further research is needed to explore engagement with wearable activity trackers over the long term (i.e. > 6 months; 27). No doubt, research into the efficacy, acceptability, and consequences of wearable activity tracking will continue to grow into the future.

## Conclusions

Findings from this study suggest that wearable activity trackers are a source of positive psychological benefits to users, including increased sense of motivation and accountability. Wearables appear to pose little risk of negative psychological consequences, although this risk is heightened in users who are low in conscientiousness or openness to experience. Given the backdrop of existing research that suggests wearables are reliable and valid devices that are effective in changing behaviour, findings from this study contribute to the growing evidence in support of wearables as a promising health behaviour change tool.

## Data Availability

The datasets used and/or analysed during the current study are available from the corresponding author on reasonable request.

## Abbreviations

F: F-test statistic
M: Mean score
N/n: Number of participants
NEO: Neuroticism, Extraversion, Openness, conscientiousness, and agreeableness
P/p: Probability value
SD: Standard deviation
SPSS: Statistical Package for the Social Sciences
t: t-test statistic
TAFE: Technical and further education
TIPI: Ten-Item Personality Inventory
